# Effects of lubabegron fumarate on ruminal fermentation and microbial community in a rumen simulation system

**DOI:** 10.3389/fmicb.2026.1863733

**Published:** 2026-06-29

**Authors:** Hongxing Zhang, Jing Xu, Xuzheng Zhou, Rongjia Han, Haiquan Li, Jiehang Li, Jingru Zuo, Yubin Bai, Weiwei Wang, Bing Li, Rongbin Hu, Jiyu Zhang

**Affiliations:** Key Laboratory of New Animal Drug Project of Gansu Province, Key Laboratory of Veterinary Pharmaceutical Development of the Ministry of Agriculture, Key Laboratory of Animal Genetics and Breeding on Tibetan Plateau, Ministry of Agriculture and Rural Affairs, Lanzhou Institute of Husbandry and Pharmaceutical Sciences of CAAS, Lanzhou, Gansu, China

**Keywords:** ammonia emissions, lubabegron fumarate, microbial protein, nitrogen utilization, ruminal fermentation

## Abstract

**Introduction:**

The expansion of the global beef industry intensifies concerns regarding ammonia emissions, posing a significant challenge to environmental sustainability and production efficiency. Lubabegron fumarate (LBF), a novel *β*-adrenergic receptor modulator approved to mitigate these emissions, lacks a defined ruminal mode of action. This study aimed to characterise the effects of LBF on *in vitro* rumen fermentation, microbial ecology, and nitrogen metabolism.

**Methods:**

Using a rumen simulation system, we evaluated the effects of LBF on fermentation parameters, nutrient digestibility, and urease activity. To elucidate the underlying mechanisms, an integrated multi-omics approach was employed to characterise shifts in microbial community structure, functional potential, and metabolic output, with a specific focus on nitrogen-cycling microbiota.

**Results:**

LBF did not directly inhibit urease. Instead, it optimised the rumen microenvironment by increasing total volatile fatty acids and pH, while reducing ammonia nitrogen (NH₃-N) and enhancing starch digestibility. Crucially, LBF orchestrated a selective enrichment of key taxa (e.g., *Ruminococcus_E*, specific *Prevotella* spp.) and promoted pivotal pathways (e.g., starch metabolism and amino acid biosynthesis) without eroding microbial diversity. Metabolomics revealed a redirected carbon flux towards propionic acid production, facilitating efficient microbial nitrogen assimilation and redirecting nitrogen from ammonia into microbial protein (MCP).

**Discussion:**

LBF reshaped the rumen microecosystem to synchronise carbohydrate fermentation and nitrogen assimilation, thereby optimising nutrient capture and reducing nitrogen waste. These findings provide a mechanistic basis for the strategic application of LBF as a pharmaceutical intervention to improve nitrogen efficiency and mitigate ammonia emissions in cattle production.

## 1 Introduction

Ammonia (NH₃) emissions from livestock production represent a significant environmental pollutant and a source of nitrogen waste, negatively impacting air quality, ecosystem health, and human health (Aneja et al., 2001; Meng et al., 2017; Thakrar et al., 2020). While dietary strategies to mitigate ruminal ammonia production are extensively studied, there is growing interest in novel pharmaceutical interventions that target host metabolism to enhance nitrogen retention and reduce excretion. Lubabegron Fumarate (LBF) is a novel *β*-adrenergic receptor modulator recently approved by the FDA as the first veterinary medicinal product specifically indicated to reduce ammonia emissions from beef cattle (FDA, 2018). Unlike traditional feed additives, LBF functions by altering host physiology. Large-scale feeding trials have demonstrated that dietary supplementation with LBF (1.5–5.5 mg/kg DM) not only reduces NH₃ emissions by up to 14.6% but also improves feed efficiency and average daily gain without compromising animal health (Kube et al., 2021; McAtee et al., 2024; Edmonds et al., 2024). Pharmacologically, LBF exhibits high affinity for *β₃*-adrenergic receptors and distinct metabolic profiles compared to conventional *β*-agonists, suggesting a favourable safety margin (Dilger et al., 2021; Hwang et al., 2022). Despite its proven *in vivo* efficacy in improving feed efficiency and reducing ammonia emissions, the underlying ruminal mechanisms of LBF remain largely unresolved. Specifically, it is unclear whether LBF directly modulates ruminal microbial fermentation or alters nitrogen metabolism within the rumen microecosystem. Elucidating these interactions is critical for optimising its application in sustainable cattle production. Given that the rumen is the central site of proteolysis and amino acid deamination, which serve as the primary sources of ammonia precursors (Firkins et al., 2007), elucidating how LBF modulates these processes is critical for optimising its application and deciphering its repartitioning mechanisms.

Therefore, this study aimed to bridge this knowledge gap by employing a rumen simulation system coupled with multi-omics approaches. We hypothesised that LBF alters ruminal fermentation patterns and reshapes the microbial community towards reduced ammonia generation. To decipher these interactions, an integrated multi-omics approach was employed to systematically evaluate the impact of LBF on ruminal fermentation profiles, microbial community structure, and metabolic signatures within a rumen simulation system. This work offers a comprehensive mechanistic understanding of the ruminal effects of LBF *in vitro*, linking pharmacological intervention to rumen microecology for improved nitrogen utilisation efficiency. Collectively, these findings provide a foundational rationale for the application of LBF in sustainable cattle production.

## 2 Materials and methods

### 2.1 Preparation of urease crude extract and inhibition assays

Rumen contents were sampled from three Simmental crossbred cattle (525.7 ± 14.36 kg, mean ± SD) before morning feeding using a rumen tube. The cattle were fed a Total Mixed Ration (TMR), with the ingredient composition and nutrient profile (dry-matter basis) detailed in Table 1. Rumen fluid from three cattle was pooled and filtered through four layers of sterile gauze. The crude urease extract was prepared as described by Zhang et al. (2025). Briefly, the filtrate was centrifuged at 300 × *g* to remove debris, followed by centrifugation at 12,000 × *g* to harvest bacterial cells. The pellet was washed twice with chilled HEPES buffer (50 mM, pH 7.5) and resuspended in 25 mL of buffer. Cells were lysed by ultrasonication (150 W, pulsed 6 s on/6 s off, 10 min) in an ice bath. The lysate was centrifuged at 13,000 × *g* for 10 min, and the resulting supernatant was used as the crude urease solution.

**Table 1:** Ingredient composition and nutrient profile of the total mixed ration (TMR) diet (dry matter basis).

Ingredient	Composition (%)	Nutrient	Content (%)
Corn silage	60.9	Crude protein	10.2
Corn	24.3	Ether extract	2.3
Wheat straw	8.7	Neutral detergent fiber	29.4
Cottonseed meal	3.2	Acid detergent fiber	14.9
Premix	1.8	Starch	39.1
Saleratus	0.9	Nitrogen-free extract	50.2
Urea	0.2		
Live yeast	0.1		

The crude urease extract was used for inhibition assays. LBF was dissolved in 10% DMSO to prepare a 400 μM stock, then diluted to working concentrations (40, 20, 10, 5, and 2.5 μM) in HEPES buffer (50 mM, pH 7.5). A solvent control containing an equivalent volume of DMSO was included. Assays were performed in 96-well plates: 40 μL of LBF solution and 40 μL of urease extract were pre-incubated at 37 °C for 10 min, followed by the addition of 100 μL of urea (25 mM) to initiate the reaction. Controls included LBF-only, urease-only, blank, and a positive control (250 μM allicin). After 30 min, reactions were terminated with potassium sodium tartrate (10 μL) and Nessler’s reagent (10 μL), and absorbance was measured at 420 nm. The formula for inhibiting urease activity is as follows:

$$\begin{array}{l} \text{Urease residual activity}\;(\%)\\ =\frac{OD_{\text{LBFgroup}/\text{allicin group}}-OD_{\text{control group}}}{OD_{\text{urease group}}-OD_{\text{urease control group}}\;}\times 100\% \end{array}$$

### 2.2 *In vitro* ruminal fermentation and sampling

*In vitro* fermentations were conducted in 100 mL anaerobic bottles sealed with rubber stoppers and aluminium caps. Each bottle contained 22 mL rumen fluid, 43 mL anaerobic medium (Menke and Steingass, 1988), 1 g of TMR (1 mm) in a fibre bag (50 μm), and 16.7 mM urea. Anaerobic conditions were established by CO₂ and reducing agents. LBF was introduced into the rumen culture medium to establish four treatments: Control (0 μM LBF), RFT (2 μM LBF), RFF (4 μM LBF), and RFE (8 μM LBF). Six replicate bottles per treatment were incubated at 39 °C and 120 rpm for 24 h. After incubation, pH was measured immediately using a pH meter (pHS-25, Shanghai Yidian Scientific Instruments Co., Ltd.). Portions of the fermentation broth were preserved with 25% metaphosphoric acid (1:1, v/v) for ammonia nitrogen (NH₃-N), urea, and volatile fatty acid (VFA) analysis, whereas separate aliquots were stored at −80 °C for MCP quantification and metagenomic and metabolomic sequencing.

After fermentation, sample bags underwent simulated small intestinal digestion (Whitehouse et al., 1994; Gargallo et al., 2006). Briefly, bags were rinsed, treated with 0.1% methylcellulose (37 °C, 30 min), and incubated in pepsin solution (1 g/L; P7000, Sigma, USA; pH 1.9, 39 °C, 1 h). Subsequently, bags were transferred to a trypsin solution (3 g/L; P-7545, Sigma, USA) prepared in 0.5 M potassium dihydrogen phosphate buffer (pH 7.75; 50 mg/L thymol) at 39 °C for 24 h. After drying at 60 °C for 48 h, nutrient digestibility was determined for pre-fermentation TMR samples and post-fermentation residues (Calsamiglia and Stern, 1995). CP was analysed via the Kjeldahl method (AOAC International, 2005), and starch was quantified using an assay kit (S0627S, Beyotime Biotechnology). Apparent digestibility was calculated as: [(Initial nutrient − Residue nutrient)/Initial nutrient] × 100%. To minimise batch-to-batch variation inherent in *in vitro* systems, all experiments were replicated on three consecutive days. Samples from the same treatment across the three days were pooled (1:1:1 ratio) prior to the analysis of routine fermentation parameters (e.g., VFA, MCP) and multi-omics profiling.

### 2.3 Measurement of ruminal fermentation parameters

MCP was quantified using the Bradford assay (P0006, Beyotime Biotechnology, China) following the modified method of Cone et al. (1997). Briefly, rumen fluid was centrifuged at 300 × *g* to remove feed particles, followed by centrifugation at 20,000 × *g* for 20 min to harvest bacterial cells. The resulting pellet was subjected to two cycles of washing with sterile saline to minimise non-microbial nitrogen contamination. Protein was extracted with 2 M NaOH at 90 °C for 10 min and neutralised with HCl. MCP concentration was determined in the supernatant using the Bradford method. For fermentation parameters, thawed samples preserved in metaphosphoric acid were centrifuged at 12,000 × g for 10 min. Urea was measured using a urease assay kit (S0574M, Beyotime Biotechnology, China). NH₃-N was determined via the phenol-hypochlorite method (Broderick and Kang, 1980). VFA was analysed using an Agilent 7,890 Gas Chromatograph equipped with a flame ionisation detector, using 4-methyl-N-valeric acid as the internal standard.

### 2.4 Metagenomic profiling of microbial composition and function

Rumen fluid samples stored at −80 °C were first pooled in a 1:1:1 ratio from six biological replicates per treatment. Subsequently, three aliquots were randomly selected from these pooled samples for analysis. Total microbial DNA was extracted using the MagBeads FastDNA Kit (MP Biomedicals, CA, USA) following the manufacturer’s instructions. DNA quantity and quality were assessed using a Qubit™ 4 Fluorometer and agarose gel electrophoresis (Sambrook and Russell, 2016). Metagenomic libraries with 400 bp insert sizes were constructed using the Illumina TruSeq Nano DNA LT Library Preparation Kit and sequenced on the Illumina NovaSeq platform (PE150) at Personal Biotechnology Co., Ltd. (Shanghai, China). Following quality filtering of raw reads using fastp, high-quality reads were assembled using MEGAHIT (contigs ≥ 300 bp) (Li et al., 2015), and open reading frames (ORFs) were predicted using Prodigal (Hyatt et al., 2010). Non-redundant gene catalogues were constructed using MMseqs2. Taxonomic profiling was conducted with Kraken2 (confidence ≥ 0.5) against the NCBI-nt database. For functional annotation, non-redundant proteins were aligned against KEGG and NCyc databases. Gene abundances were quantified by mapping reads to the gene catalogue using Strobealign and counting with featureCounts. Differential abundance analysis was performed using LEfSe with a default LDA score threshold of >2.0, a widely accepted cutoff indicating a biologically relevant effect size (Segata et al., 2011). Visualisation via volcano plots utilised standard thresholds of FDR < 0.05 and |log₂ fold change| > 1. Principal component analysis (PCA) was performed on variance-stabilised transformed abundances to visualise overall variation. Beta diversity based on Bray–Curtis dissimilarity was visualised via principal coordinate analysis (PCoA).

### 2.5 Non-targeted metabolomic profiling and data analysis

Non-targeted metabolomics was performed on the same samples used for metagenomic analysis. Metabolites were extracted from 300 μL rumen fluid using a methanol:acetonitrile (1:1, v/v) solvent system, vacuum-dried, and reconstituted in 50% methanol containing 2-chlorophenylalanine (internal standard) (Want et al., 2010). Chromatographic separation was performed on an ACQUITY UPLC HSS T3 column (40 °C) using a 0.4 mL/min gradient elution, coupled to a Thermo Orbitrap Exploris 120 mass spectrometer. Raw data were processed using MS-DIAL for peak picking, alignment (Tsugawa et al., 2015), and metabolite identification via MS/MS spectral matching against mzCloud, LIPID MAPS, and HMDB libraries. Features detected in < 50% of QC samples were filtered, and data were log-transformed and Pareto-scaled prior to multivariate analysis (PCA, OPLS-DA) using MetaboAnalyst 5.0 (Pang et al., 2021). Model validity was confirmed via 200-permutation testing, and analytical reproducibility was ensured by QC RSD values < 20%.

### 2.6 Statistical analysis

Statistical analyses were performed using R (v4.3.2) and GraphPad Prism (v10.0, GraphPad Software, USA), with data visualised using the ggplot2 package. Data are presented as mean ± standard error of the mean (SEM). Group differences were assessed using the Kruskal-Wallis test, followed by Dunn’s post-hoc test with false discovery rate (FDR) correction. Associations between rumen microbiota and fermentation parameters were evaluated using Spearman correlation analysis, and the collective influence of physicochemical factors on microbial structure was assessed via redundancy analysis (RDA). Statistical significance was defined as FDR-adjusted *p* < 0.05.

## 3 Results

### 3.1 Impact of LBF on *in vitro* ruminal fermentation parameters

**Figure 1:**
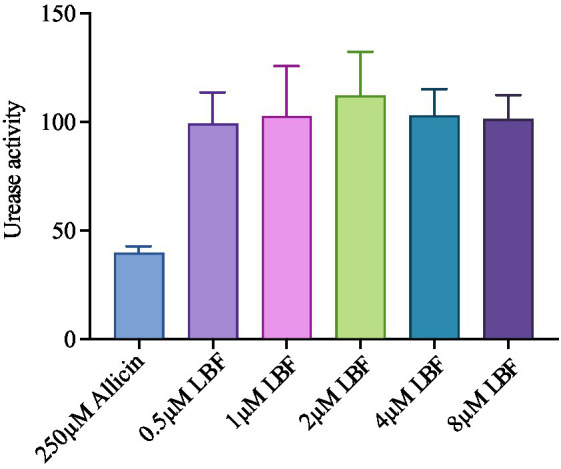
Effects of allicin and LBF at different concentrations on urease activity.

**Figure 2:**
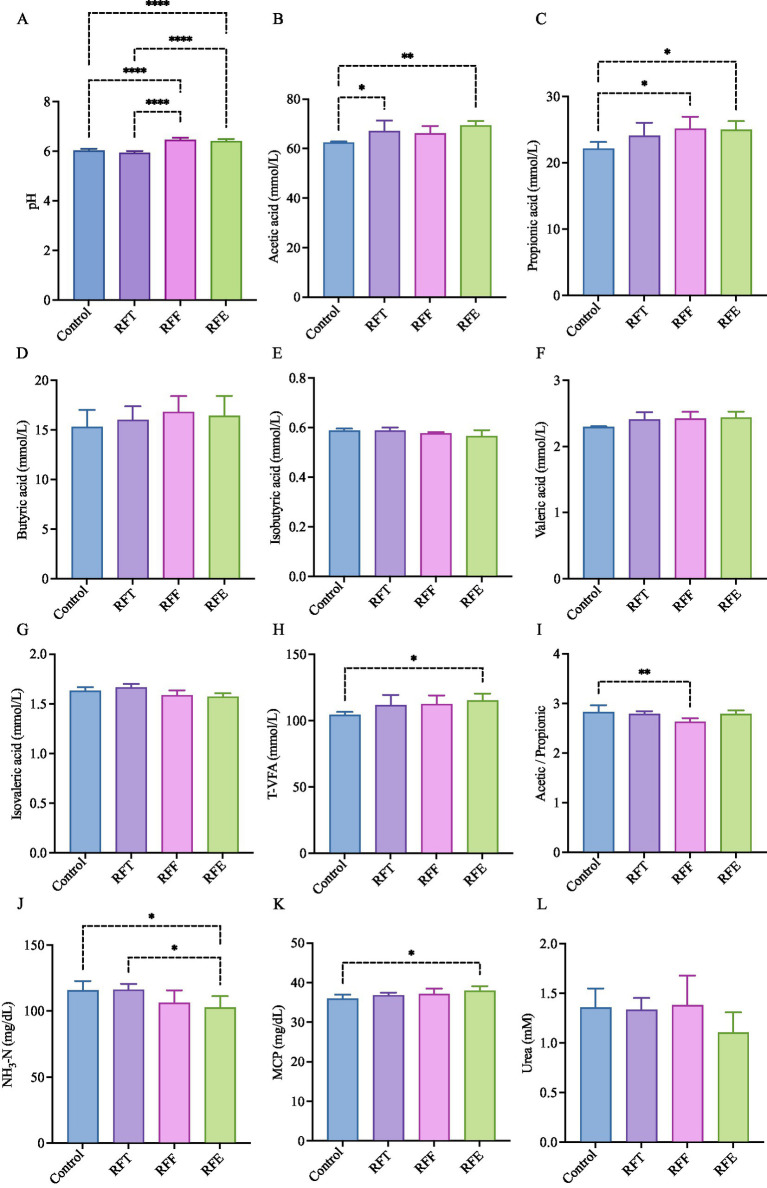
Effects of different treatments on ruminal fermentation parameters *in vit*ro. Bar graphs **(A–L)** illustrate profiles including pH, VFA, and other parameters. Data are presented as mean ± SEM (*n* = 6). Symbols above the bars (e.g., *) indicate statistically significant differences between groups (*p < 0.05, **p < 0.01, ***p < 0.001, ****p < 0.0001).

As shown in Figure 1, allicin (250 μM) served as a positive control, decreasing relative urease activity to approximately 40%. Conversely, LBF treatments at concentrations ranging from 2 to 8 μM did not inhibit urease activity. Based on the lack of direct urease inhibition by LBF at concentrations up to 8 μM in the isolated enzyme assay, doses of 2, 4, and 8 μM were selected for the subsequent *in vitro* rumen fermentation trial. Compared to the control, LBF treatments altered rumen fermentation profiles (Figure 2). Both RFF and RFE significantly increased ruminal pH (*p* < 0.0001). RFE treatment also elevated total volatile fatty acid (TVFA) concentrations (*p* < 0.05). Regarding VFA composition, RFT and RFE increased acetic acid concentrations (*p* < 0.05 and *p* < 0.01, respectively), whereas RFF and RFE increased propionic acid concentrations (*p* < 0.05 and *p* < 0.01, respectively). Consequently, the acetic acid-to-propionic acid ratio was significantly lower in the RFF group than in the control (*p* < 0.01). In terms of nitrogen metabolism, RFE treatment reduced NH₃-N concentrations (*p* < 0.05) while increasing MCP yields (*p* < 0.05). No significant differences in urea concentrations were observed among the groups.

### 3.2 Dose-dependent effects of LBF on nutrient digestibility

**Figure 3:**
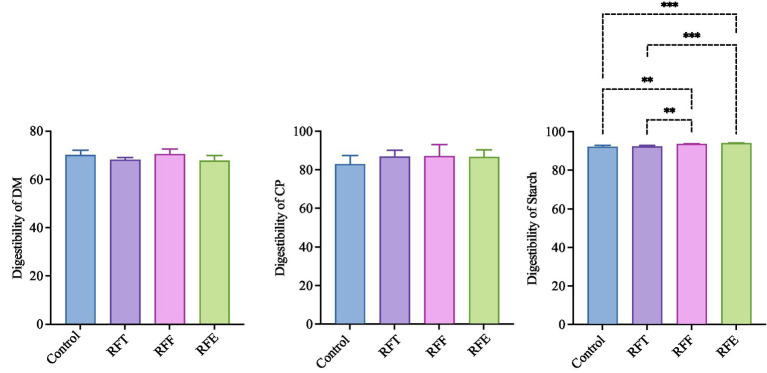
Effects of LBF concentration on dry matter, crude protein, and starch digestibility. Symbols above the bars (e.g., *) indicate statistically significant differences between groups (*p < 0.05, **p < 0.01, ***p < 0.001, ****p < 0.0001).

Dry matter and crude protein digestibility remained stable across all treatments, averaging approximately 70% and approximately 80%, respectively (*p* > 0.05; Figure 3). In contrast, starch digestibility increased with LBF treatment. RFE increased starch digestibility to 94% compared to the control (92%; *p* < 0.001), and RFF also elevated it to 94% (*p* < 0.01). No significant difference was observed between RFT (~93%) and the control (*p* > 0.05).

### 3.3 Ruminal microbiota composition

#### 3.3.1 Taxonomic composition and functional potential

**Figure 4:**
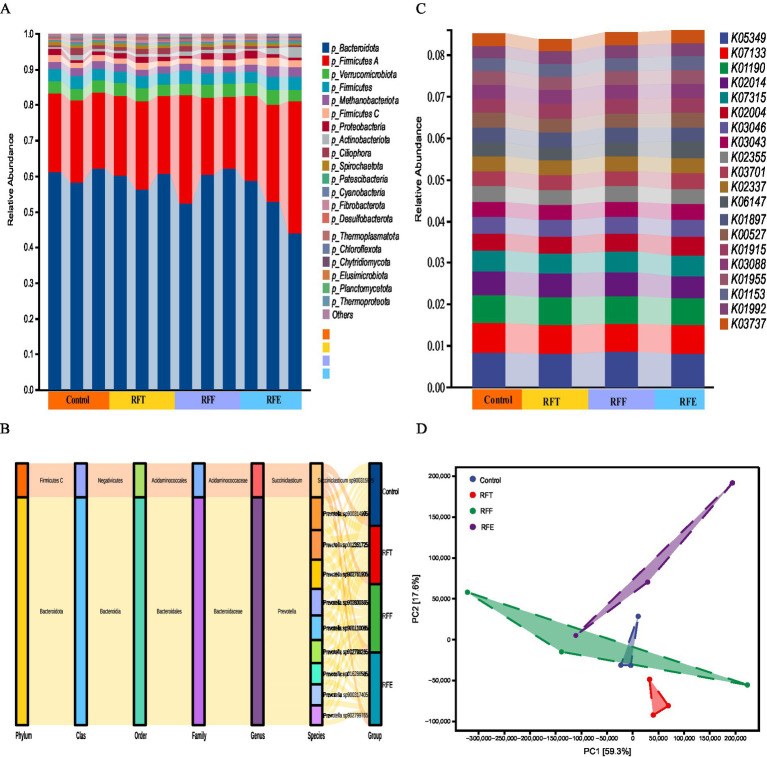
*In vitro* rumen microbial community profiling across treatments. **(A)** Phylum-level taxonomic distribution. **(B)** Taxonomic composition at different taxonomic levels. **(C)** Relative abundance of the top 20 KEGG Orthology (KO) across treatment groups. **(D)** Principal component analysis (PCA) of microbiota profiles.

The rumen microbial community structure and functional potential were evaluated across treatment groups (Figure 4). At the phylum level (Figure 4A), *Bacteroidota* and *Firmicutes* predominated across all groups, constituting the majority of the community. At the genus level, *Prevotella* was the dominant taxon. Taxonomic progression from phylum to species revealed a consistent correlation: high *Bacteroidota* abundance corresponded with high prevalence of *Prevotella* and its associated species. The distribution of specific microbial taxa varied among the treatment groups. At the species level (Figure 4B), *Prevotella* exhibited treatment-specific enrichment: *Prevotella sp002351725* and *sp902761905* were predominant in RFT; *sp900110085* and *sp902800365* characterised RFF; and *sp016286585*, *sp902799765*, and *sp900317405* were representative of RFE. In contrast, *Succiniclasticum sp900315925* was most abundant in the Control group. Functional profiling of the top 20 KEGG Orthology (KO) categories (Figure 4C) revealed high abundance of core metabolic pathways across all groups, particularly carbohydrate, amino acid, and energy metabolism (e.g., KO5349 and KO1190). Principal component analysis (PCA) demonstrated clear separation among treatment groups (Figure 4D). The control, RFT, RFF, and RFE groups formed distinct clusters, with PC1 and PC2 explaining 59.3 and 17.6% of the total variance, respectively.

#### 3.3.2 *β*-diversity and community structure

**Figure 5:**
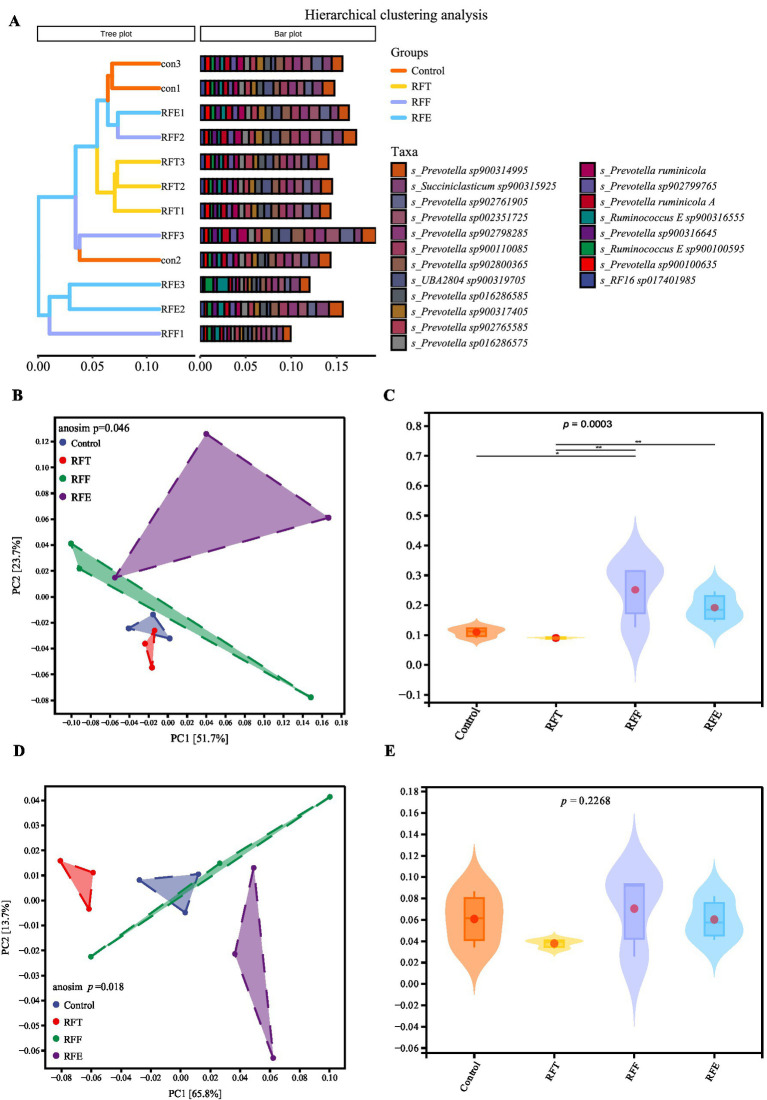
Integrated analysis of microbial community structure and functional profiles. **(A)** Hierarchical clustering and taxonomic composition of microbial communities across treatment groups. **(B,C)** Principal Coordinate Analysis (PCoA) of community structure based on Bray–Curtis dissimilarity. **(D,E)** PCoA of functional profiles based on Bray–Curtis dissimilarity. Differences among groups along principal coordinates were tested by the Kruskal-Wallis test. Asterisks denote statistical significance (*p < 0.05, **p < 0.01, ***p < 0.001, ****p < 0.0001).

Alpha diversity indices for both taxonomy and function showed no significant differences among treatments (*p* > 0.05; Supplementary Figures 1, 2), indicating stable within-sample diversity. In contrast, beta diversity analysis revealed distinct structural shifts among treatment groups (Figure 5). Hierarchical clustering analysis (Figure 5A) demonstrated that samples from the same treatment clustered tightly together, forming distinct branches that were clearly separated from the Control and other treatment groups. PCoA ordination visually confirmed these findings, revealing significant separation in both taxonomic composition (ANOSIM, *p* = 0.046; Figure 5B) and functional potential (ANOSIM, *p* = 0.018; Figure 5D). Notably, the RFE treatment occupied a distinct position in the ordination space, exhibiting the most pronounced deviation from the Control group in both profiles. Consistent with the structural shifts, the microbial community composition exhibited significant alterations (*p* = 0.0003; Figure 5C). In contrast, the overall functional profiles of the microbial communities showed no significant difference (*p* = 0.2268; Figure 5E), suggesting that the core metabolic potential was preserved despite the changes in specific taxonomic abundances.

#### 3.3.3 Differential abundance and functional analysis

**Figure 6:**
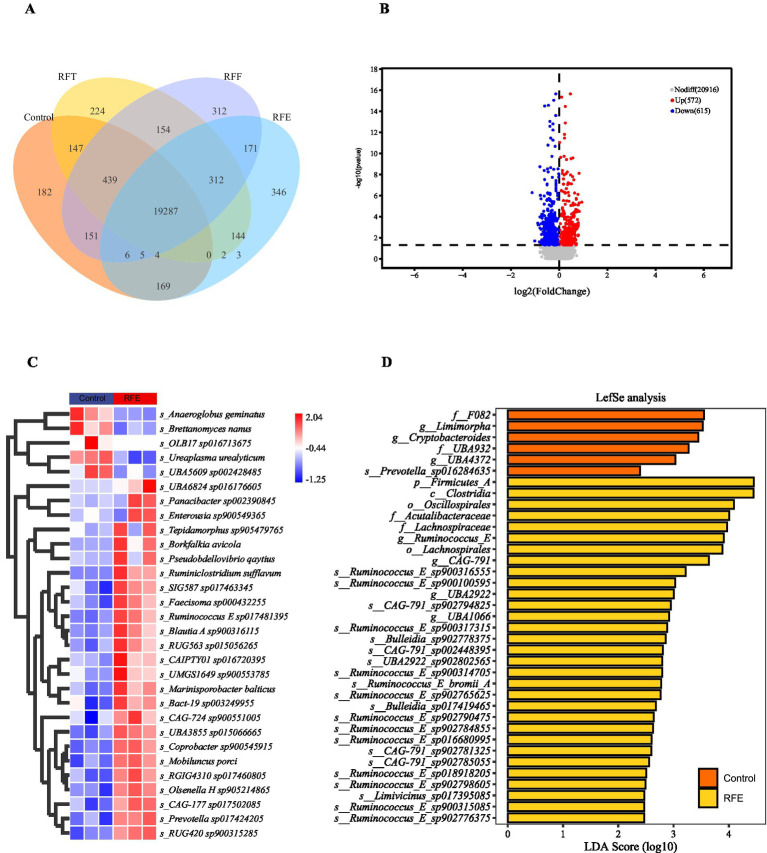
Multi-faceted analysis of microbial community differences among treatment groups. **(A)** Venn diagram showing the core and unique microbiota across the Control, RFT, RFF, and RFE groups. **(B)** Volcano plot comparing the differential abundance of species between the Control and RFE groups. Species that are significantly upregulated (red) or downregulated (blue) in the RFE group compared to the Control group are highlighted. **(C)** Heatmap visualising the relative abundance of selected bacterial species in Control and RFE samples. **(D)** Linear Discriminant Analysis Effect Size (LEfSe) bar graph identifying bacterial clades with significant differential abundance.

Shared and unique species among groups were revealed via Venn diagram (Figure 6A). A comparative analysis between the Control and the most divergent RFE group demonstrated significant restructuring. The volcano plot (Figure 6B) identified 1,187 differentially abundant species, with 572 upregulated and 615 downregulated in RFE. Hierarchical clustering (Figure 6C) confirmed a clear separation, highlighting the enrichment of specific taxa (e.g., *s_Prevotella_sp017424205, s_RUG420_sp900315285*) in RFE, while others (e.g., *s_Anaeroglobus geminatus*, *s_Brettanomyces nanus*) were enriched in the Control group. LEfSe analysis (Figure 6D) revealed specific biomarkers, such as the genera *Limimorpha* and *Cryptobacteroides* for the Control group, and the genera *Ruminococcus_E*, *CAG-791, and UBA1066* for RFE. Functional differential analysis (Supplementary Figure 3) corroborated these taxonomic shifts. The volcano plot (Supplementary Figure 3A) revealed significant alterations in 106 KOs, with 63 upregulated (e.g., K17264) and 43 downregulated (e.g., K00101) in RFE. LEfSe analysis further identified specific metabolic pathways discriminative for RFE, including amino acid metabolism, carbohydrate metabolism (e.g., starch and sucrose metabolism), and cofactor biosynthesis (Supplementary Figure 3B).

#### 3.3.4 Correlation networks between microbiota and phenotypes

**Figure 7:**
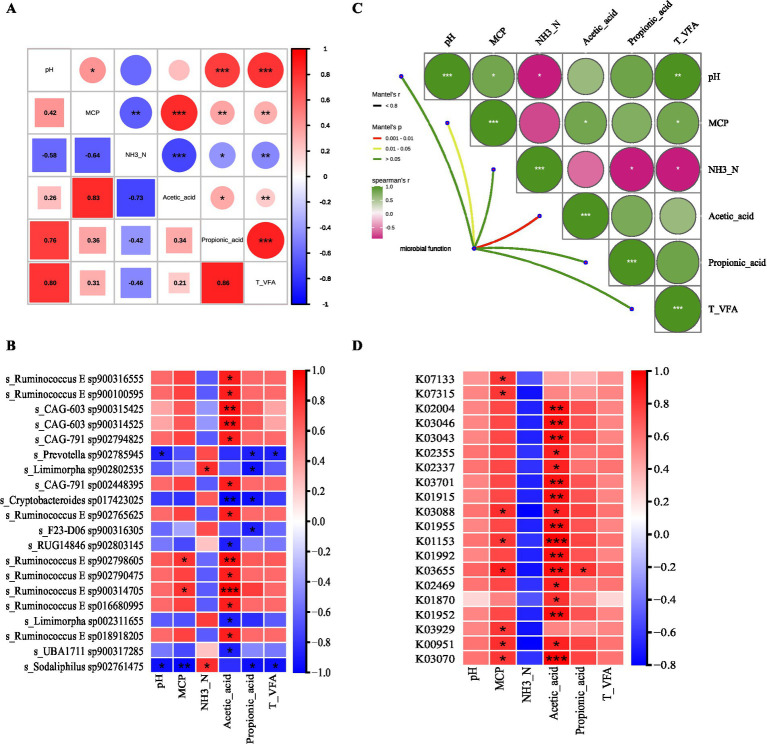
Multi-panel analysis of correlations among fermentation parameters, microbial communities, and functions. **(A)** Heatmap of pairwise correlation coefficients among fermentation parameters. Color intensity indicates correlation strength and direction. **(B)** Heatmap of Pearson correlations between bacterial species abundance and fermentation parameters. **(C)** Circos plot visualising the correlation matrix between microbial functions and fermentation parameters. **(D)** Heatmap of Pearson correlations between bacterial species abundance and KEGG orthologs (KOs). Asterisks indicate statistical significance (*p < 0.05, p** < 0.01, p*** < 0.001, and ****p < 0.0001).

Pairwise correlation analysis revealed distinct association patterns among fermentation parameters (Figure 7A). Strong positive correlations were observed within the VFA cluster (TVFA, acetic acid, and propionic acid). Specifically, pH was positively correlated with total MCP (r = 0.42, *p* < 0.05) and TVFA (r = 0.80, *p* < 0.01), whereas MCP was negatively correlated with NH₃-N (r = −0.64, *p* < 0.01). Pearson correlation analysis linked specific taxa to these phenotypes (Figure 7B). *Ruminococcus_E* spp. (e.g., *sp900316555*, *sp900314705*) showed significant positive correlations with acetic acid and MCP. In contrast, *Prevotella sp902785945* and *Sodaliphilus sp902761475* were negatively correlated with TVFA and pH. A Circos plot visualised the functional linkages (Figure 7C), mapping key microbial functional modules to fermentation parameters. Notably, specific modules were concurrently correlated with both MCP and acetic acid, highlighting their central metabolic roles. Direct correlations between fermentation parameters and individual KOs were further validated (Figure 7D).

### 3.4 Impact of LBF on nitrogen-cycling microbiota

#### 3.4.1 Diversity and composition of nitrogen-cycling bacteria

Alpha diversity of nitrogen-cycling microbial communities remained stable across all treatments, with no significant differences observed in either taxonomic or functional diversity indices (*p* > 0.05; Supplementary Figures 4, 5, respectively). Rarefaction and rank-abundance curves confirmed consistent sampling depth and species distribution patterns among groups. In contrast, beta diversity analysis revealed significant restructuring of the nitrogen-cycling microbiota (Supplementary Figure 6). Hierarchical clustering grouped samples by treatment, driven primarily by shifts in the relative abundance of key genera such as *Prevotella* (Supplementary Figure 6A). PCoA ordination confirmed significant separation in both taxonomic composition (*p* = 0.0463; Supplementary Figures 6B,C) and functional potential (*p* = 0.0079; Supplementary Figures 6D,E). These results demonstrate that LBF supplementation selectively enriched specific nitrogen-cycling guilds, thereby reconfiguring community structure without altering core diversity.

#### 3.4.2 Differential abundance of nitrogen-cycling bacteria and functional potential

Volcano plot analysis (Supplementary Figure 7A) revealed pronounced shifts in species abundance between the Control and RFE groups, with 17 species significantly upregulated and 18 downregulated in the RFE group (*p* < 0.05). LEfSe analysis (Supplementary Figure 7B) further identified the specific bacterial clades characterising each treatment. The RFE group was primarily enriched with several *Ruminococcus_E* species (e.g., *sp900317315 and sp900314565*) and specific members of the genus *Prevotella* (e.g., *Prevotella_sp902764705*). Conversely, the Control group was predominantly associated with distinct microbial taxa, including the phylum Spirochaetota (along with its derived class Spirochaetia, order Sphaerochaetales, and family Sphaerochaetaceae) as well as specific species such as *Cryptobacteroides_sp002371115* and *Breznakia_sp001695645*. Notably, certain *Prevotella* species (*sp902765585*, *sp902775605*, and *sp902800245*) were the dominant biomarkers characterising the Control group. Comparative functional profiling revealed a divergence in nitrogen metabolism strategies between the two groups (Supplementary Figure 7C). While the Control group exclusively enriched the ferredoxin-nitrite reductase module, the RFE group consolidated a suite of four co-enriched modules comprising assimilatory nitrate reductase, NADH-dependent nitrite reductase, urease, and glutamine synthetase. This configuration indicates that the microbial community evolved into a state of enhanced nitrogen conservation, actively channelling available nitrogen into microbial biomass synthesis rather than allowing its accumulation as free ammonia. These functional shifts provide a mechanistic explanation for the restructured microbial community and the improved nitrogen economy observed *in vitro*.

### 3.5 LBF modulates the *in vitro* fermentation metabolome

#### 3.5.1 Differential metabolite profiling

**Figure 8:**
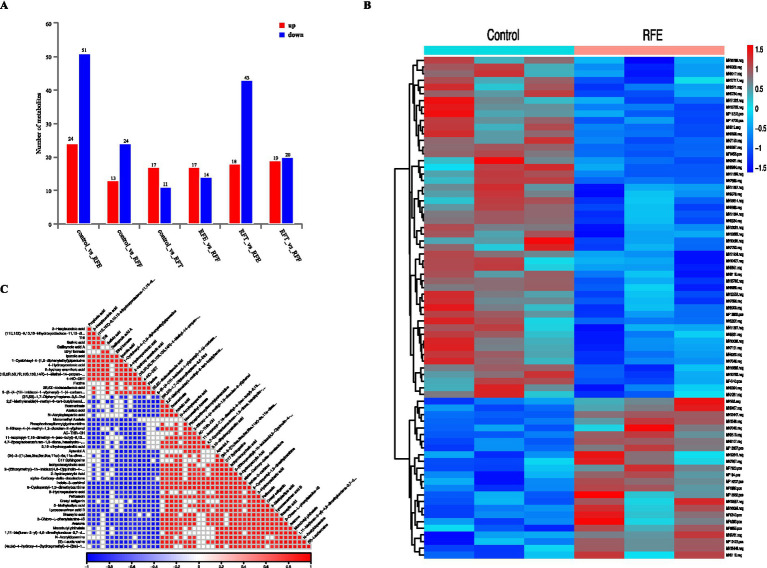
Multi-level analysis of differential metabolites among treatment groups. **(A)** Bar plot showing the number of significantly upregulated (red) and downregulated (blue) metabolites in comparisons among treatment groups. **(B)** Heatmap displaying the abundance profiles of differential metabolites across samples from the Control and RFE groups. **(C)** Correlation heatmap of differential metabolites. Pairwise correlations are shown; blue and red indicate negative and positive correlations, respectively.

Quality control validation confirmed the high reproducibility of the analytical platform (r = 1.00; Supplementary Figure 8A). PCA revealed distinct metabolic separations among the Control, RFT, RFF, and RFE groups (Supplementary Figure 8C), indicating that each treatment induced profound alterations in the metabolic profile. As illustrated in Supplementary Figures 9A,B, lipids and lipid-like molecules constituted the predominant metabolite class across both ionisation modes. Comparative analysis further demonstrated that the RFE group induced the most extensive metabolic reprogramming, with a total of 75 differentially regulated metabolites identified relative to the Control group (Figure 8A). Notably, this metabolic shift was predominantly characterised by a widespread downregulation of metabolites. Orthogonal Projections to Latent Structures Discriminant Analysis (OPLS-DA) further corroborated the robust separation between the Control and RFE groups (Supplementary Figure 9C), demonstrating that RFE exposure systematically reshaped the metabolomic profile.

#### 3.5.2 Metabolite correlation and pathway analysis

**Figure 9:**
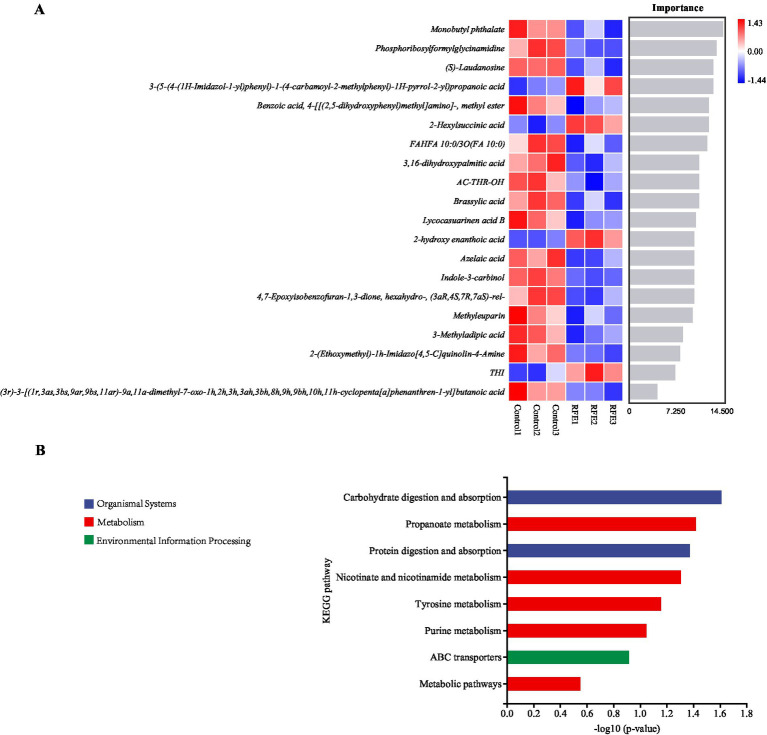
Analysis of discriminatory metabolites and enriched biological pathways. **(A)** Differential heatmap showing the abundance profiles of key discriminatory metabolites, which were ranked by their importance scores in a Random Forest model for group discrimination. **(B)** Pathway enrichment analysis of differential metabolites (RFE vs. Control).

A heatmap of differential metabolites confirmed a clear segregation between the Control and RFE groups, highlighting coordinated clusters of up-regulated and down-regulated features (Figure 8B). Correlation network analysis further revealed a significant rewiring of metabolic associations in the RFE group, characterised by distinct modules of co-expressed metabolites (Figure 8C). Notably, propionic acid exhibited strong positive correlations with key organic acids (e.g., 2-hexylsuccinic acid, sativic acid, ipurolic acid, 4-hydroxycrotonic acid, and 2-hydroxy acid), suggesting a coordinated shift in central carbon metabolism. Random Forest ranking identified specific metabolites, including propionic acid and 2-hydroxyenanthoic acid, as top discriminators between groups (Figure 9A). Pathway enrichment analysis demonstrated that these alterations were primarily mapped to core metabolic categories, significantly enriching pathways related to carbohydrate digestion and absorption, propionic acid metabolism, and protein digestion and absorption (Figure 9B). This integrated analysis demonstrates that RFE treatment drove fundamental shifts in microbial energy harvest and short-chain fatty acid biosynthesis.

## 4 Discussion

### 4.1 Modulation of ruminal fermentation profile by LBF

Although *in vivo* studies report that LBF reduces ammonia emissions (FDA, 2018; Edmonds et al., 2024), the underlying mechanism remains unclear. It was initially hypothesised that LBF might directly inhibit urease, a key enzyme in urea hydrolysis. To establish physiologically relevant testing conditions, the *in vitro* concentration was extrapolated from reported effective dietary doses (13–90 mg per head per day). Based on a reported rumen contents volume (∼57 L) in adult beef cattle (Church et al., 1993), the theoretical intraruminal concentration of LBF was estimated to be approximately 4 μM, assuming uniform distribution within the rumen fluid. First, assay validity was confirmed via the urease inhibition assay using the urease inhibitor allicin (Zhang et al., 2025). However, inhibition assays demonstrated that LBF exerted no suppressive effect on urease activity at concentrations up to 8 μM. Instead, lacking evidence of direct enzymatic targeting, the mechanism appears to involve systemic regulation of the rumen microbiota. The RFE treatment elicited the most comprehensive improvements in fermentation efficiency. Concurrent elevations in pH, total VFA, and MCP yield, coupled with a significant reduction in NH₃-N, indicate a repartitioning of nitrogen flow from excessive ammonia toward microbial protein synthesis, representing a hallmark of enhanced nitrogen utilisation efficiency (Hristov et al., 2013; Wei et al., 2023). Given the stability of crude protein digestibility, this enhanced nitrogen utilisation efficiency appears to stem from improved ammonia assimilation by specific microbial guilds rather than altered proteolysis. Furthermore, RFE induced a strategic shift in carbon metabolism. The marked increase in acetic acid and propionic acid yield culminated in an elevated TVFA yield, reflecting a broad stimulation of key fermentative pathways. Notably, the high starch digestibility (~94%) suggests a mechanistic connection to the succinate-propionic acid pathway, similar to the effect of fumarate supplementation (Baldwin et al., 1962; Ungerfeld and Kohn, 2006). Fumarate functions as a key intermediate and electron acceptor in the rumen, selectively driving the succinate-propionic acid pathway (Araújo et al., 2011; Remling et al., 2014). This suggests that LBF favours microbial guilds specialised in starch fermentation and propionic acid synthesis, thereby linking enhanced energy harvest to efficient nitrogen retention. A limitation of this study is the lack of direct measurements of gas production kinetics and methane emissions. Future work should integrate continuous gas recording to fully resolve how LBF modulates ruminal gas profiles and methanogenesis, especially considering the observed shifts in the acetic acid/propionic acid (A/P) ratio.

### 4.2 LBF reshaped the ruminal microbial community *in vitro*

The phenotypic improvements in fermentation were accompanied by restructuring of the rumen microbiota (Fuhrman, 2009). LBF treatments, particularly RFE, modulated the ruminal ecosystem by selectively shifting *β*-diversity without compromising *α*-diversity. This suggests that LBF induced a targeted compositional restructuring that was functionally significant despite unaltered overall diversity. The enrichment of *Prevotella* and *Ruminococcus_E* in the RFE group provided a microbial basis for the observed metabolic shifts. Specifically, the selective amplification of these taxa correlated with enhanced starch digestibility and propionic acid yield, consistent with their established roles in carbohydrate metabolism and cross-feeding (Bekele et al., 2010; Seshadri et al., 2018). Functional analysis corroborated this link, revealing that RFE induced upregulation of KEGG pathways governing carbohydrate uptake and amino acid biosynthesis (Taxis et al., 2015). This metabolic rewiring optimised the existing microbial machinery, with enhanced substrate scavenging fuelling energy harvest and increased precursor flux bolstering MCP synthesis (Allison, 1969). Consequently, RFE reshaped a functional consortium that efficiently partitioned starch carbon toward propionic acid and channelled nitrogen into microbial biomass, thereby reducing metabolic losses. Correlation networks mapped this functional shift to specific drivers, with strong positive associations among TVFA, acetate, and propionic acid confirming coordinated fermentative activity. Crucially, the elevated pH alongside high VFA indicated enhanced acid buffering capacity, likely attributable to proton consumption during ammonium assimilation into MCP. This mechanism is directly supported by the strong negative correlation between MCP and NH₃-N. In summary, LBF functioned as a selective force that reshaped the rumen ecosystem toward a new homeostasis. By amplifying keystone taxa and their metabolic pathways, LBF enhanced the synchronised capture of carbon and nitrogen, driving the improved fermentation efficiency observed *in vitro*. It is important to note that direct evidence linking LBF to specific ruminal microbial mechanisms remains limited; existing studies have largely characterised its effects on animal growth and on ammonia-related outcomes. Therefore, the present study provides a novel, data-driven multi-omics framework for identifying the ruminal microbial and metabolic pathways through which LBF may exert its effects. We acknowledge that these inferences remain speculative. While quantitative PCR (qPCR) would provide absolute quantification of these key taxa, this study focused on characterising system-level shifts through high-throughput sequencing and multi-omics integration. Future targeted *in vitro* and *in vivo* experiments, including qPCR validation, are required to confirm these taxonomic shifts.

### 4.3 LBF reshapes nitrogen-cycling microbial community and function *in vitro*

Nitrogen use efficiency depends on microbial community composition rather than mere gene abundance (Wang et al., 2024; Yan et al., 2026). This principle guided our analysis of the nitrogen cycling microbiota following LBF treatment. The observed reduction in NH₃-N and increase in MCP highlighted improved nitrogen efficiency. Alpha diversity remained stable across treatments, indicating that LBF preserved the microbial functional reservoir rather than acting as a broad antimicrobial. However, beta diversity analysis revealed significant structural reconfigurations, particularly in the RFE group. RFE selectively enriched *Ruminococcus_E* (e.g., *sp900317315*) and *Prevotella* (e.g., *sp902764705*) species. Modern genomics confirms these taxa possess extensive peptidase and amino acid catabolic enzymes (Betancur-Murillo et al., 2023). Crucially, these genera exhibit efficient ammonia transport systems and rapidly assimilate ammonium ions into amino acids for biomass synthesis (Wang et al., 2019). Their enrichment suggests efficient channelling of amino acids into microbial biomass instead of ammonia accumulation. This microbial assimilation, rather than physicochemical pH effects or direct urease inhibition, is the primary biological mechanism driving the observed NH₃-N reduction in the RFE group. While *Ruminococcus_E* is classically fibrolytic, its production of acetate and hydrogen provides critical energy substrates (Greening et al., 2019). This bridges fibre degradation and nitrogen assimilation, creating a syntrophic linkage between carbon and nitrogen cycles (Ungerfeld, 2020). Functional profiling substantiated this mechanism, revealing divergent nitrogen-metabolism modules between groups. In contrast to the Control group, which exclusively enriched the ferredoxin-nitrite reductase module, the RFE group consolidated a complete assimilatory cascade comprising nitrate reductase, NADH-nitrite reductase, urease, and glutamine synthetase, thereby channelling oxidised nitrogen and recycled urea into biomass and minimising ammonia accumulation (Kim et al., 2017). Consequently, LBF optimised existing pathways rather than introducing new functions. By amplifying keystone taxa and suppressing inefficient competitors, LBF tightened the coupling between energy-yielding fermentation and nitrogen anabolic demand, as conceptualised in modern models of rumen metabolic networking (Mizrahi et al., 2021). This targeted remodelling of the rumen ecosystem explains the improved nitrogen economy observed *in vitro*.

### 4.4 Modulation of ruminal metabolomic profiles by LBF

Untargeted metabolomics revealed the biochemical phenotype driven by LBF, with PCA and OPLS-DA models showing the profound metabolic reprogramming unique to RFE. This shift featured coordinated organic acid regulation, specifically 2-hexylsuccinic acid. This metabolite acts as a biomarker of the succinate pathway, confirming that RFE drives *Ruminococcus_E* and *Prevotella* enrichment to enhance propionic acid synthesis (Greening et al., 2019; Ungerfeld, 2020). Pathway enrichment linked metabolite shifts to macroscopic phenotypes, validating enhanced starch digestibility and propionic acid production via propanoate metabolism. Crucially, combined enrichment of protein digestion forged a biochemical link to nitrogen economy, confirming efficient microbial assimilation over ammonia accumulation. This assimilation, fuelled by ATP from propionic acid synthesis, elevated nitrogen demand and retention efficiency (Mizrahi et al., 2021; Du et al., 2025). LBF elevated succinate propionate flux and protein assimilation, altering the metabolite pool to enrich specific organic acids and create a positive feedback loop that stabilised the high-efficiency state (Zeng et al., 2019). Collectively, metabolomics confirmed that LBF remodelled the community into a coordinated network, wherein elevated carbon flux toward propionic acid and enhanced nitrogen assimilation tightened the coupling between energy harvest and protein synthesis. This targeted reprogramming enabled LBF to optimise the ecosystem, thereby achieving superior fermentation efficiency.

## 5 Conclusion

This study demonstrates that LBF, particularly the RFE group, improves rumen fermentation efficiency via ecological remodelling rather than direct enzymatic inhibition. Multi-omics integration revealed that RFE induced targeted restructuring of the microbial community. This process selectively enriched keystone taxa such as *Ruminococcus_E* and *Prevotella* while preserving overall taxonomic and functional diversity, indicating precise modulation rather than a broad antimicrobial effect. Mechanistically, RFE elevated carbon flux through the succinate propionate pathway, evidenced by increased propionate and specific metabolites like 2-hexylsuccinic acid. Concurrently, upregulated amino acid biosynthesis fuelled microbial anabolism. The energy from enhanced fermentation drove efficient nitrogen capture into microbial protein, reducing ammonia and improving nitrogen retention. Thus, LBF optimised resource allocation to achieve synchronised carbon and nitrogen metabolism. Therefore, this study provides a mechanistic foundation for applying LBF to mitigate nitrogen emissions in farming.

## Data Availability

The metagenomic sequencing data generated in this study have been deposited in the NCBI Sequence Read Archive (SRA) under BioProject accession PRJNA1478094. Associated metabolomics data are publicly available in MetaboLights under accession number MTBLS14756. Additional experimental data and analysis scripts supporting this work are hosted on GitHub at https://github.com/dr-zhang-qj/RF-V0-LBF.
