# Supplementary material for: Effects of lubabegron fumarate on ruminal fermentation and microbial community in a rumen simulation system
Source: Front Microbiol. 2026 Jun 29;17:1863733. doi: 10.3389/fmicb.2026.1863733 (PMC13361298; doi:10.3389/fmicb.2026.1863733)
Supplement: Supplementary file 1 [file Data_Sheet_1.PDF]

Supplementary Material

1 Supplementary Figures and Tables

1.1 Supplementary Figures

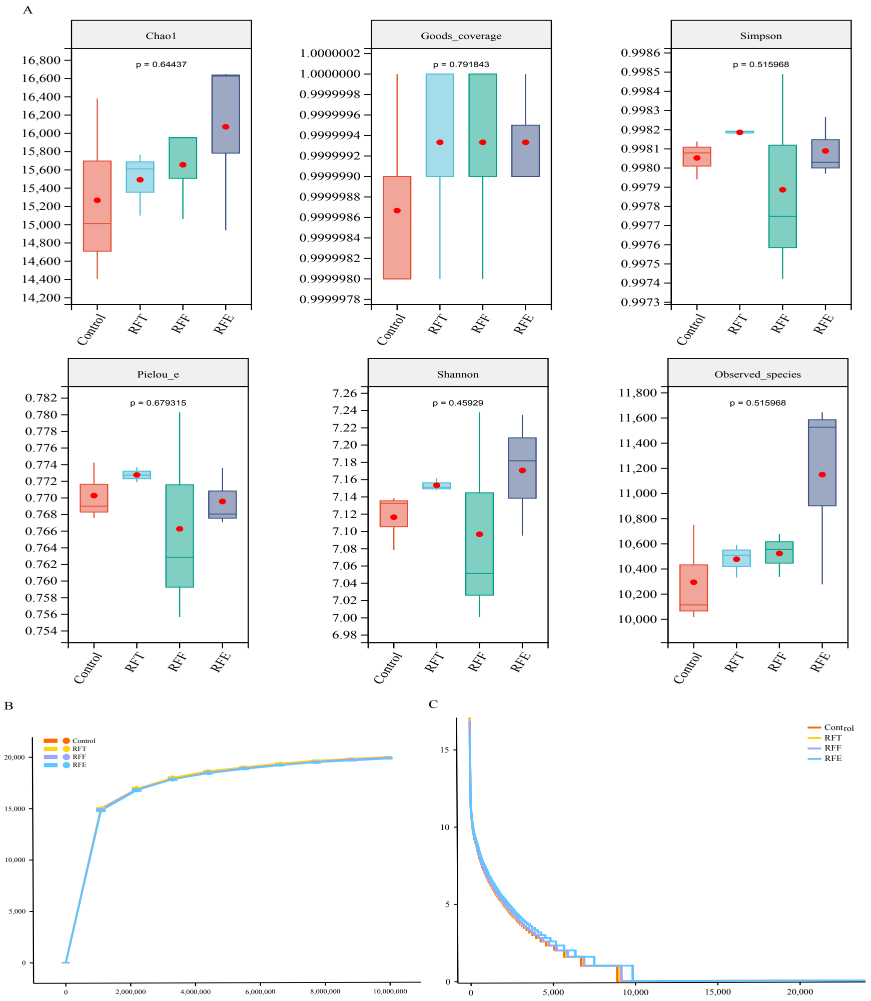

Supplementary Figure 1. Microbial diversity and richness indices among different treatment groups. (A) Box plots showing the distribution of alpha diversity indices (Chao1, Goods coverage, Simpson, Pielou's evenness, Shannon, and Observed species) for the four experimental groups (Control, RFT, RFF, and RFE). The center line in each box represents the mean. *p*-values indicate no significant differences among the groups for any index. (B) Rarefaction curves of nitrogen-cycling microbial communities. (C) Rank-abundance curves of microbial communities.

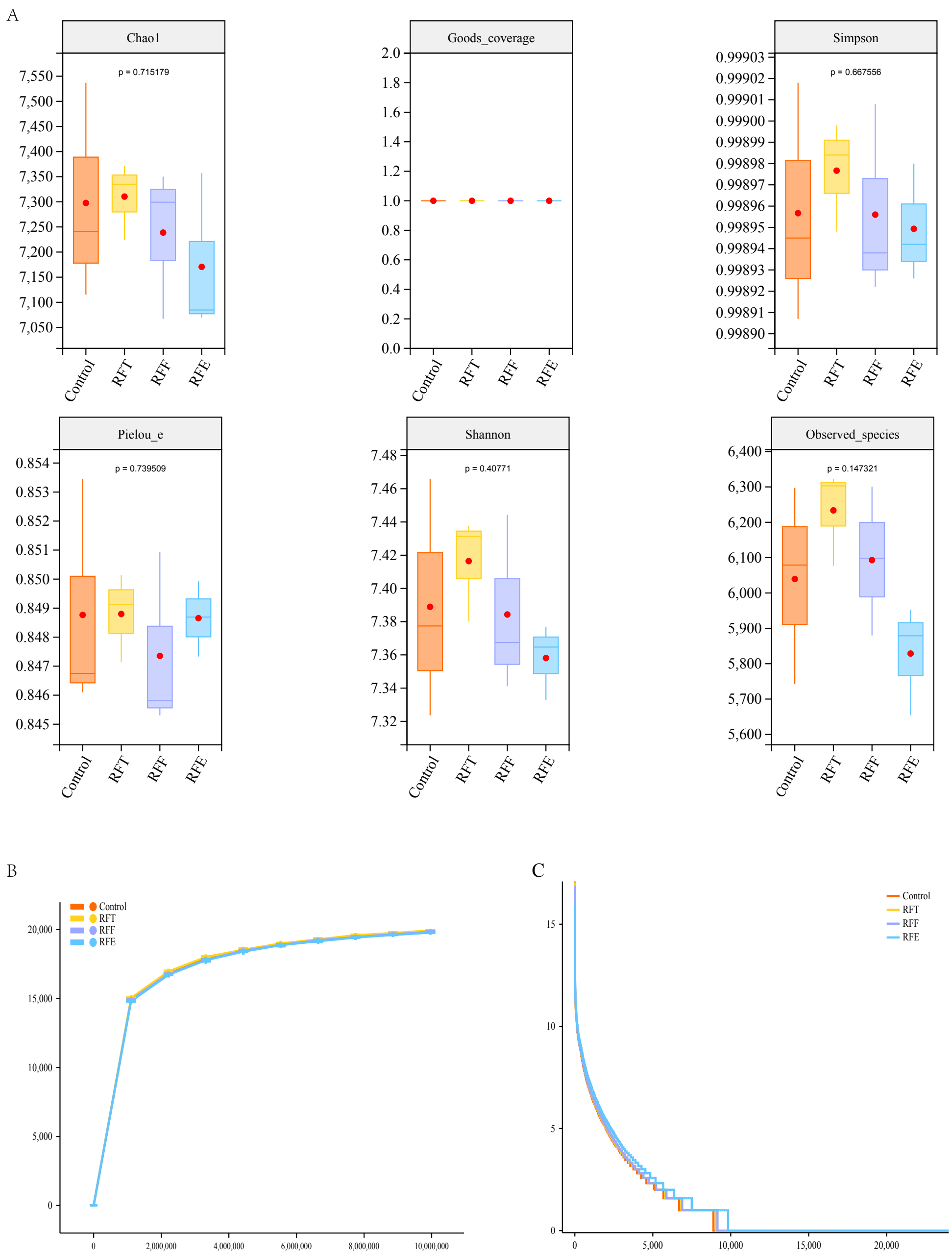

Supplementary Figure 2. Functional diversity and gene detection sufficiency among treatment groups based on KEGG enrichment. (A) Box plots comparing functional alpha diversity (Chao1, Goods coverage, Simpson, Pielou's evenness, Shannon, and Observed species) among the four experimental groups (Control, RFT, RFF, and RFE). No significant differences ($p > 0.05$) were observed for any index. (B) Rarefaction curves of KEGG orthologs (KOs) for each experimental group. (C) Rank-abundance curves of KOs among treatment groups.

A B

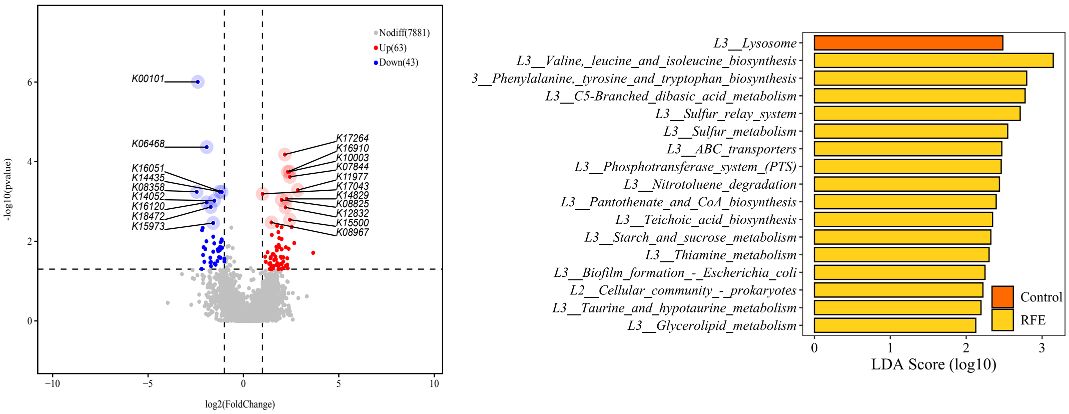

Supplementary Figure 3. Integrated analysis of differential microbial functions between the Control and RFE groups. (A) Volcano plot highlighting significantly differential KOs between the Control and RFE groups. (B) Linear Discriminant Analysis Effect Size (LEfSe) bar graph identifying enriched functional biomarkers in Control and RFE.

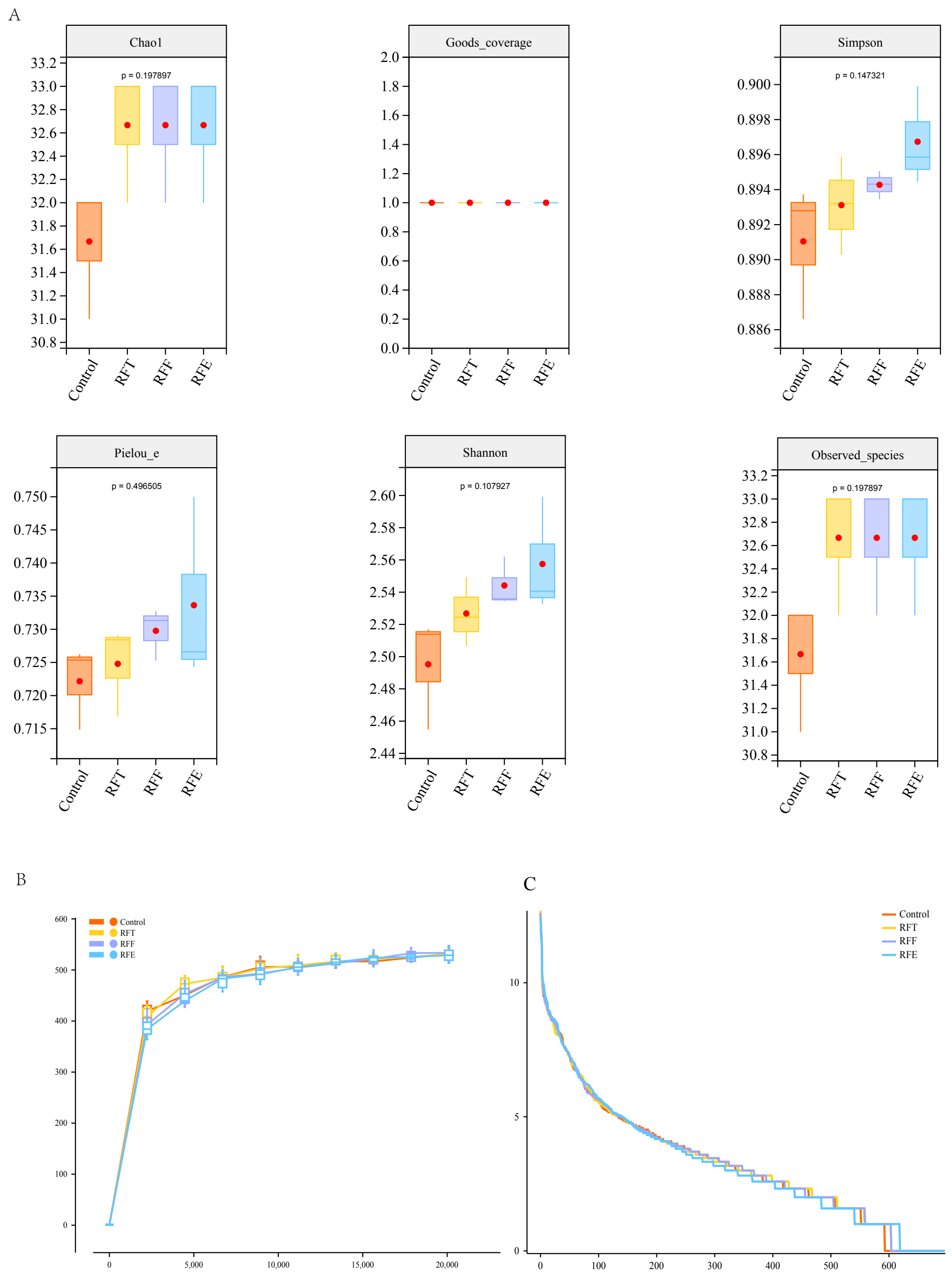

Supplementary Figure 4. Alpha diversity of nitrogen-cycling microbial communities among experimental groups. (A) Box plots showing the distribution of alpha diversity indices (Chao1, Goods coverage, Simpson, Pielou's evenness, Shannon, and Observed species) for the four experimental groups (Control, RFT, RFF, and RFE). The center line in each box represents the mean. No significant differences ($p > 0.05$) were observed among the groups for any index. (B) Rarefaction curves of nitrogen-cycling microbial communities. (C) Rank-abundance curves of nitrogen-cycling microbial communities.

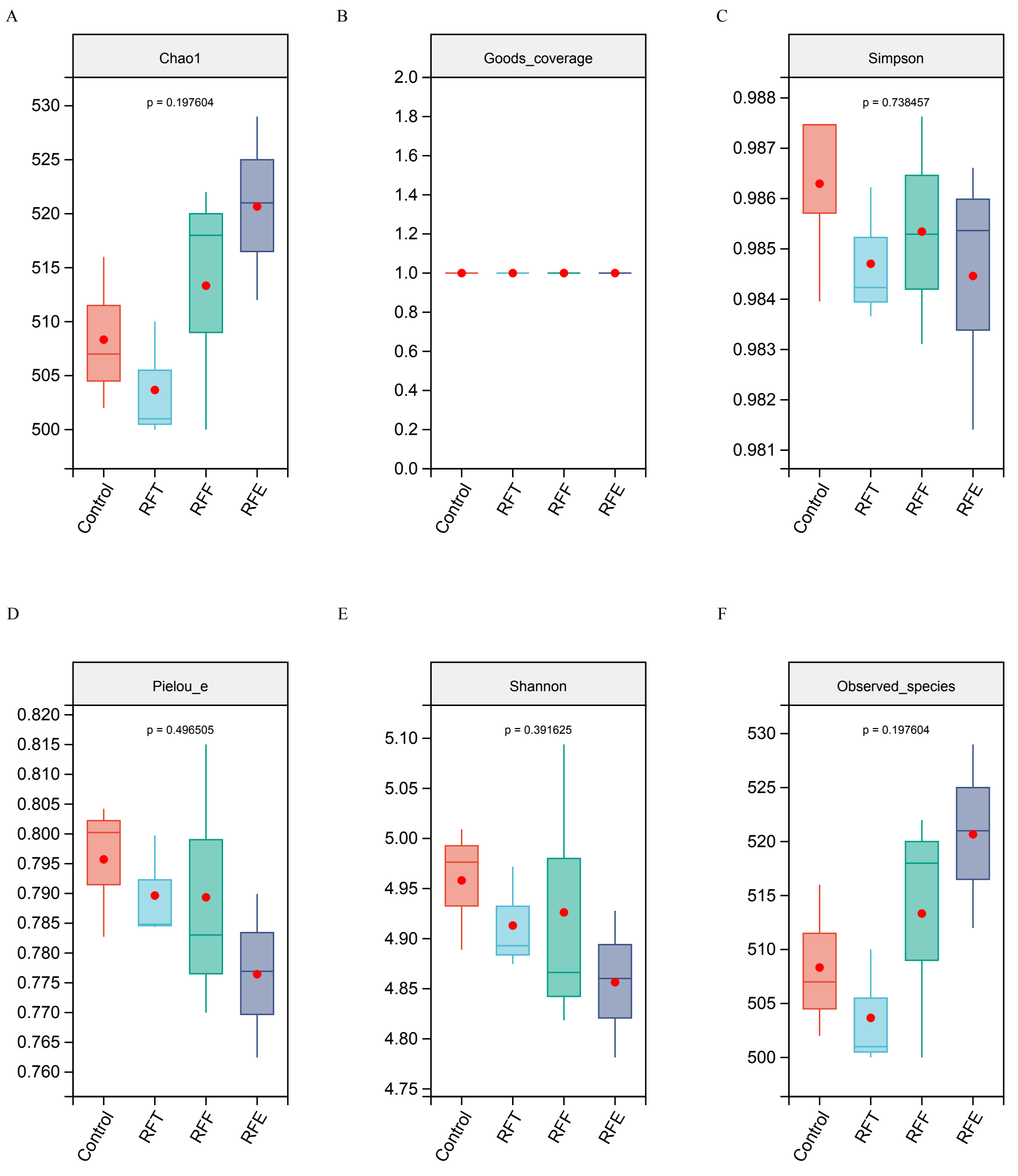

Supplementary Figure 5. Functional diversity among treatment groups based on nitrogen-cycle-related KEGG orthologs. (A-F) Box plots comparing functional alpha diversity (Chao1, Goods coverage, Simpson, Pielou's evenness, Shannon, and Observed species) among the four experimental groups (Control, RFT, RFF, and RFE). No significant differences (p > 0.05) were observed for any index.

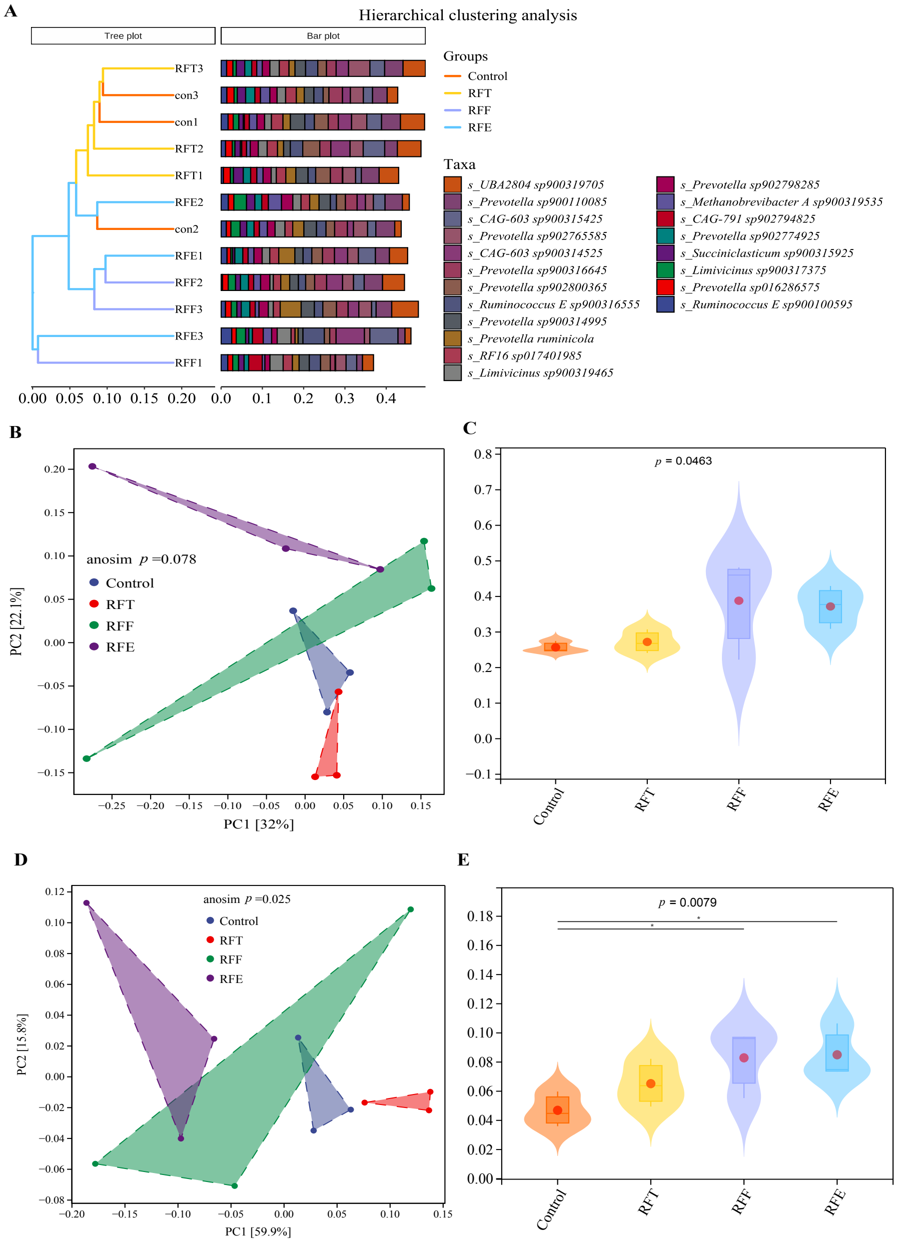

Supplementary Figure 6. Community structure and functional profiling of nitrogen-cycling microorganisms. (A) Hierarchical clustering and taxonomic composition of nitrogen-cycling microbial communities among treatment groups. (B, C) Principal Coordinate Analysis (PCoA) of community structure based on Bray-Curtis dissimilarity. (D, E) PCoA of functional profiles based on Bray-Curtis dissimilarity. Differences among groups along principal coordinates were tested by the Kruskal-Wallis test. Asterisks denote statistical significance (*$p < 0.05$, **$p < 0.01$, ***$p < 0.001$, *** *$p < 0.0001$).

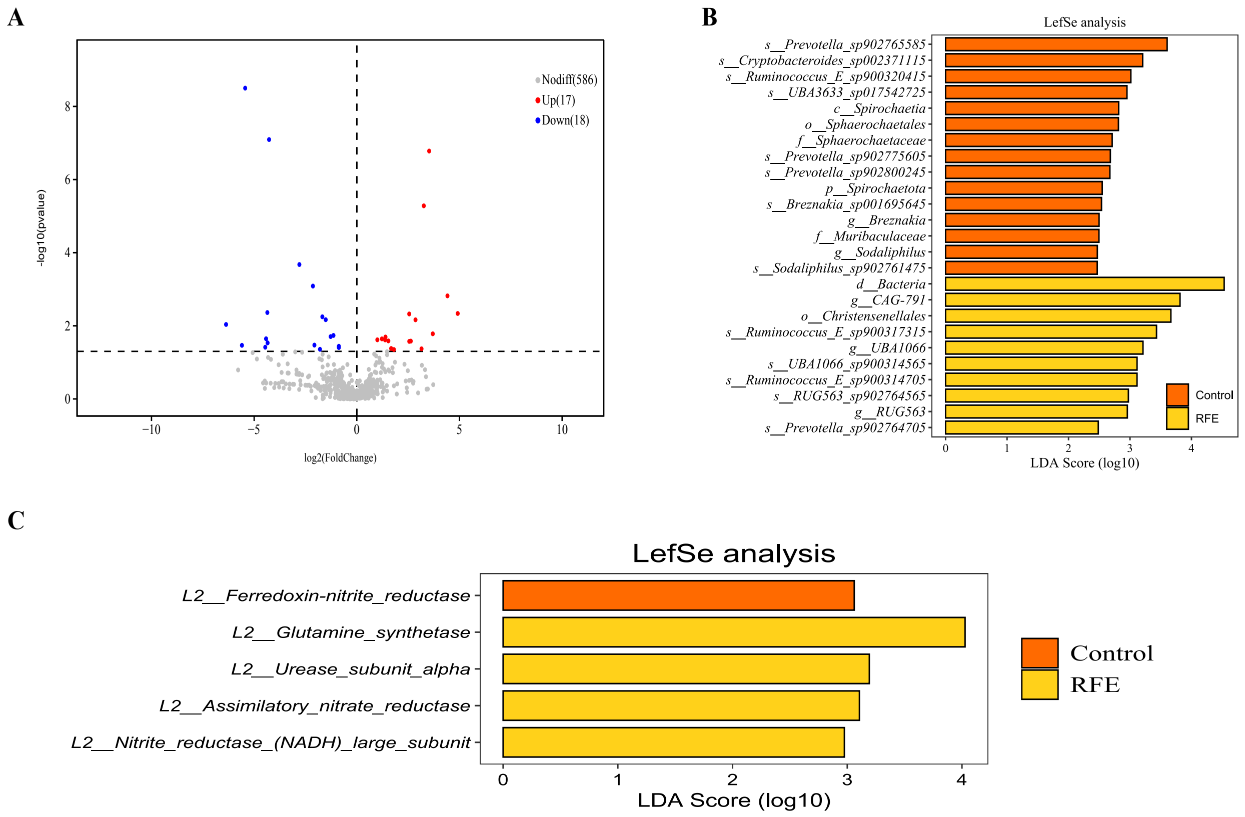

Supplementary Figure 7. Multi-faceted analysis of nitrogen-cycling microbial community differences between the Control and RFE groups. (A) Volcano plot comparing the differential abundance of species between the Control and RFE groups. Species that are significantly upregulated (red) or downregulated (blue) in the RFE group compared to the Control group are highlighted. (B) Linear Discriminant Analysis Effect Size (LEfSe) bar graph identifying bacterial clades with significant differential abundance. (C) LEfSe bar graph identifying enriched functional biomarkers in Control and RFE.

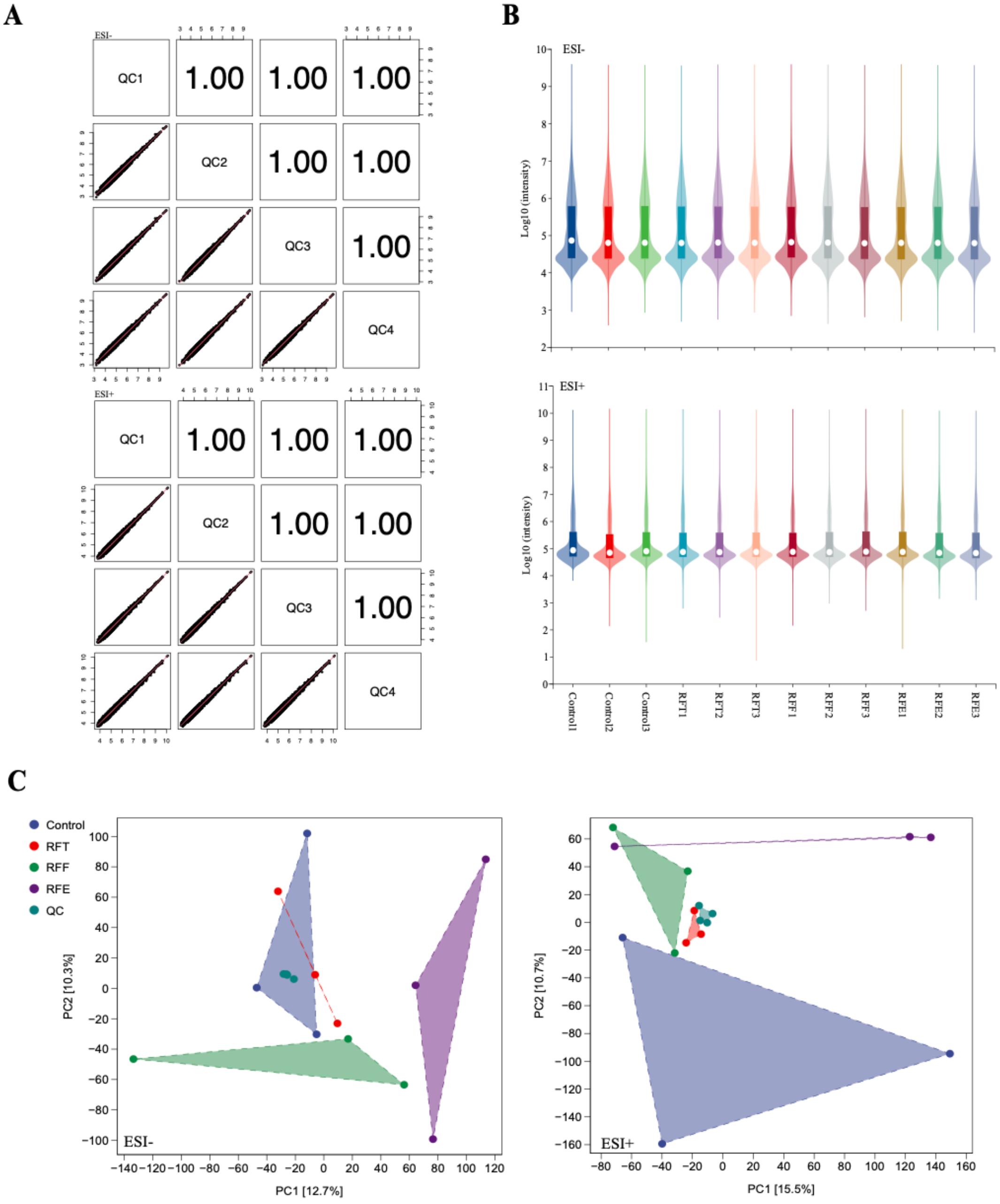

Supplementary Figure 8. Assessment of metabolomic data quality and overview of sample clustering. (A) Correlation matrix of QC samples showing perfect reproducibility (r = 1.00). (B) Distribution of feature intensities in ESI- and ESI+ modes. (C) Principal component analysis (PCA) score plots illustrating the separation of treatment groups (Control, RFT, RFF, RFE) in both ionization modes.

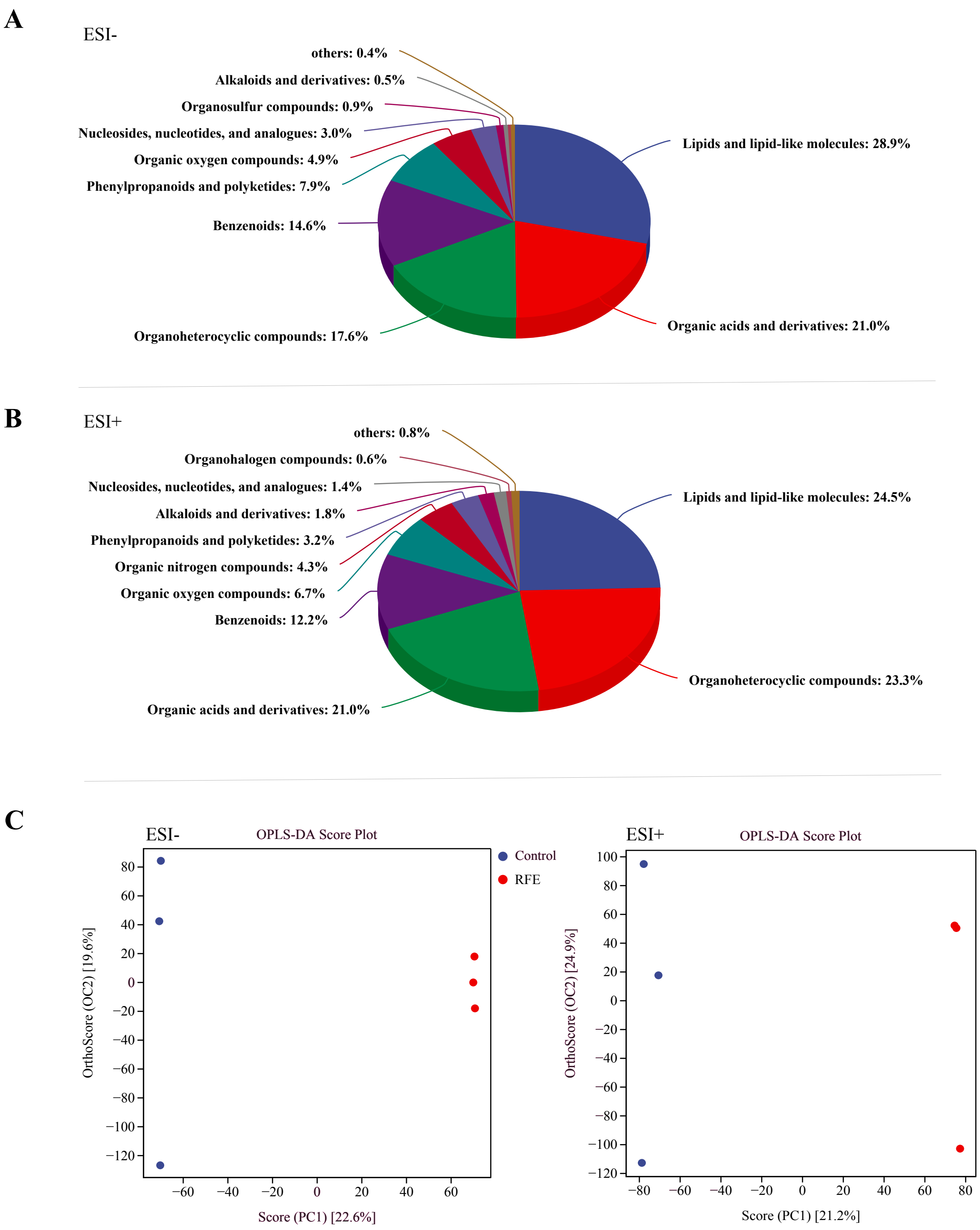

Supplementary Figure 9. Overview of metabolomic composition and supervised separation between Control and RFE groups. (A, B) Pie charts illustrating the relative percentage abundance of major metabolite classes identified in the (A) ESI-negative (ESI-) and (B) ESI-positive (ESI+) ionization modes. (C) Orthogonal Projections to Latent Structures Discriminant analysis (OPLS-DA) score plots derived from the (left) ESI- and (right) ESI+ mode data, respectively.
